# Virtual Grid Engine: a simulated grid engine environment for large-scale supercomputers

**DOI:** 10.1186/s12859-019-3085-x

**Published:** 2019-12-02

**Authors:** Satoshi Ito, Masaaki Yadome, Tatsuo Nishiki, Shigeru Ishiduki, Hikaru Inoue, Rui Yamaguchi, Satoru Miyano

**Affiliations:** 1 The Institute of Medical Science, The University of Tokyo, Shirokanedai 4-6-1, Minato-ku, Tokyo, 108-8639 Japan; 2 Frontier Computing Center, Fujitsu Limited, Higashishinbashi1-5-2, Minato-ku, Tokyo, 105-7123 Japan

**Keywords:** High performance computing, Grid engine, TOP500, MPI, Python

## Abstract

**Background:**

Supercomputers have become indispensable infrastructures in science and industries. In particular, most state-of-the-art scientific results utilize massively parallel supercomputers ranked in TOP500. However, their use is still limited in the bioinformatics field due to the fundamental fact that the asynchronous parallel processing service of Grid Engine is not provided on them. To encourage the use of massively parallel supercomputers in bioinformatics, we developed middleware called *Virtual Grid Engine*, which enables software pipelines to automatically perform their tasks as MPI programs.

**Result:**

We conducted basic tests to check the time required to assign jobs to workers by VGE. The results showed that the overhead of the employed algorithm was 246 microseconds and our software can manage thousands of jobs smoothly on the K computer. We also tried a practical test in the bioinformatics field. This test included two tasks, the split and BWA alignment of input FASTQ data. 25,055 nodes (2,000,440 cores) were used for this calculation and accomplished it in three hours.

**Conclusion:**

We considered that there were four important requirements for this kind of software, non-privilege server program, multiple job handling, dependency control, and usability. We carefully designed and checked all requirements. And this software fulfilled all the requirements and achieved good performance in a large scale analysis.

## Introduction

The use of supercomputers in bioinformatics has become common with the unprecedented increase in the amount of biomedical data, e.g., DNA sequence data, and also demands of complex data analysis using multiple software tools. The growth of data size has been due to drastic improvements of measurement devices in the last decade. For DNA sequence data, the speed of data generation and reduction of the cost were over-exponential due to the development of so-called Next-Generation Sequencers (NGSs) [1].

DNA analyses of variant diseases have been estimated to require tens of thousands of sample analyses [2]. Furthermore, the size of sample such as read length and coverage tends to be larger rapidly. However, only a few studies have utilized massively parallel supercomputers ranked in TOP500 [3–6]. One of the main reasons is lack of *Grid Engine* (GE) services, e.g., *Sun Grid Engine* and *Univa Grid Engine*, on most of those supercomputers; the use of GE-like service is currently almost a prerequisite for large-scale biological data analyses..

Most software and programs that run on such TOP500-like supercomputers are paralleled using Message Passing Interface (MPI)[7], wherein all subprocesses work synchronously. On the other hand, array jobs, automatically paralleled subprocesses of software pipelines by GE are asynchronous. Therefore, the GE conflicts with MPI-based systems from the perspective of the job-filling factor.

Here, the MPI parallelization of software pipelines requires expert knowledge and experience. It is necessary for the MPI parallelization of software pipelines to use C or Fortran language wrapper programs or to commission High Performance Computing (HPC) experts to overwrite them fundamentally, which will be difficult for users.

Recently, Cloud-base systems, such as Amazon Web Services (AWS), have been popular in NGS data analysis [8]. Cloud computing services are very useful for small laboratories and companies that do not have computational resources. However, they still require significant costs for large-scale analyses [9]. In addition, there are still several problems to be overcome, such as data transfer time, data corruption checking, and data security management.

From the perspective of HPC, DRAGEN [10] achieved drastic acceleration of the GATK pipeline [11]. The hardware implementation of all the processes in GATK using FPGA caused this great acceleration. This approach is the ultimate software-tuning technique. On the other hand, it makes it quite difficult to improve the implemented workflows. GATK is one of the most popular pipelines for germline mutation calling, so this tuning is extremely efficient for it.

However, there is a great variety of target variants for NGS data analyses for each study, and it is inevitable for algorithms and pipelines to be designed for the study. Therefore, general software pipelines still have merits in many studies and massively paralleled supercomputers are useful for accelerating their analyses.

In this study, we developed MPI-based middleware named *Virtual Grid Engine* (VGE) that enables software pipelines based on GE system to run on massively parallel supercomputers.

## Implementation

### Goal and requirements

Supercomputers are always used by many users. Thousands of jobs from users will be submitted to the system. Required system resource such as calculation time, necessary nodes, memory size, etc., are varies from job to job. Therefore, job management system (JMS) that controls the assignment of jobs efficiently is inevitable for large scale supercomputers.

There are some JMS and GE-like tools. *Sun Grid Engine*, *Univa Grid Engine*, and *TORQUE* [12] are pure GE systems. *GNU parallel* enables users to employ commands or software in parallel [13]. While those GE-like tools are useful, it is a problem that not all of the supercomputer system do not implement them. That has prevented to analyze biological data on those massively parallel supercomputer systems.

GE is a kind of server service program that works with JMS. JMS is one of the core service programs of supercomputers, so that a user cannot install or change it on a target machine. Thus, it is extremely hard for bioinformatics pipelines to work on a supercomputer that does not equip any GE.

Our goal is to perform user pipelines efficiently on supercomputers on which GE has not been installed. VGE is the first application for this field, which must be a user program and act like a server service. To achieve this objective, there are the following four requirements: 1. Non-privilege server program VGE must be a user program and also works as a server service program. In order to utilize thousands of CPUs, it must be a MPI parallel program, and also provide a job submission mechanism for user pipelines. 2. Multiple job handling VGE must accept a number of jobs (many samples, multiple type analyses, etc.) simultaneously. Most MPI programs running on large-scale supercomputers often use thousands to tens of thousands of processes; thus running a job with a small number of processes a dozen times is inefficient. 3. Dependency control VGE must handle dependencies among tasks. Tasks in pipelines often contain dependencies, meaning that a certain task must wait for the former task to be accomplished. 4. Usability VGE must be friendly for medical and bioinformatics researchers. For example, file formats and programming languages must be widely used in this research area, and the modification of user pipelines for using VGE must be minimal. This is the most important point.It must be noted that software that does not satisfy this requirement will have severe problems in their dissemination.

### Algorithms

**Fig. 1:**
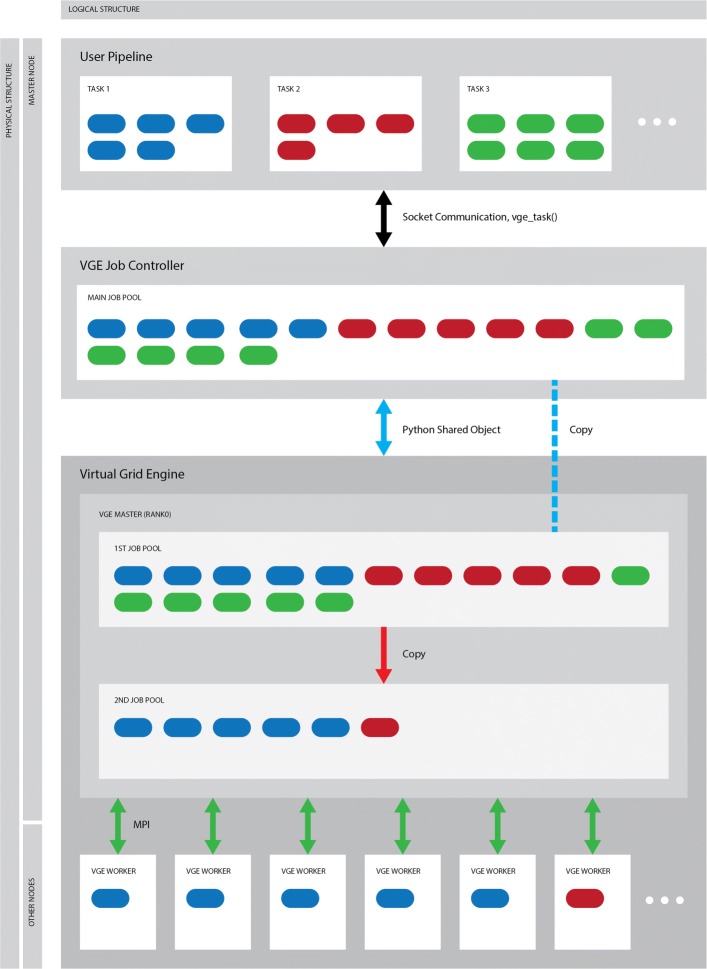
VGE system

To satisfy the most important requirement of the system, that is, the fourth requirement, VGE was written in Python. We also employed the *MPI4PY* package for the MPI environment of Python [14–16]. The system uses a Master-Worker model, which enables it to accept jobs with different processing details or different number of processes concurrently. Figure 1 shows the concept of the VGE system. The VGE system consists of two programs: a Master-Worker type MPI paralleled main program (VGE: Virtual Grid Engine) and a job controller program (VGE job controller) that controls the job information between the VGE and user pipelines.

Job submission is performed by *vge_task()* function in place of GE commands, such as *qsub* of *SGE*. Scripts for submission and the number of array jobs are passed to *vge_task()* as arguments. *vge_task()* then sends the job information to VGE using socket communication.

The information is stored in the main job pool of VGE on the Python shared object. The VGE master process, rank 0 of VGE MPI processes, plays this role, assigns registered jobs in the main pool to sleeping workers for execution, then waits for the completion signal from workers.

The VGE job controller uses two types of communication: the socket communication of the *vge_task()* function, and the Python *shared object* for the main job pool. User pipelines, the VGE job controller, and the VGE maser process must be executed on the same physical computer unit (master node) to allow these two types of communication.

The key point in the master-worker algorithm is the overhead cost for assigning jobs to workers. The master node executes three processes: the user pipeline, VGE job controller, and VGE master process. However, the former two processes only perform the registration of jobs to VGE, and their calculation loads are negligible.

Therefore, the computational cost of the VGE master for assigning jobs to workers is critical for the performance of this system. Its cost is closely related to the access frequency to the main job pool and its size.

To overcome these two problems, the VGE master creates two local job pools in itself. One pool (the second job pool) is used for reducing the access frequency by copying all jobs from the main job pool to it at a certain time (the blue dashed line in Fig. 1). The other pool (the first job pool) extracts jobs equal to the number of VGE workers from the second job pool (the red arrow in Fig. 1).

By assigning jobs from the first job pool, VGE reduces the size of the job pool to access and minimize its overhead.

## Results

In this section, we conducted several tests to check the performance of VGE. As described above, the VGE basic performance depends on the overhead time for assigning jobs. Therefore, we first checked this using an elementary code with a number of array jobs. Then, we performed a large-scale analysis test using practical data.

### Overhead measurements

Here, we conducted basic tests to check the time required to assign jobs to workers by VGE. The test code comprised only 120 seconds of sleep. The numbers of array jobs were 10,000 (Case 1) and 100,000 (Case 2). The numerical environment was 2000 nodes of the K computer [17]. Table 1 shows the specification of the K computer. VGE used a node for the master process, so the number of workers was 1999.

**Table 1:** Specifications of the K computer

CPU	SPARC64 VIIfx 2.0 GHz 8 cores/socket
RAM	16 GB/node (2GB/core)
Node	82,944
Capability	10,510 TFlops

**Fig. 2:**
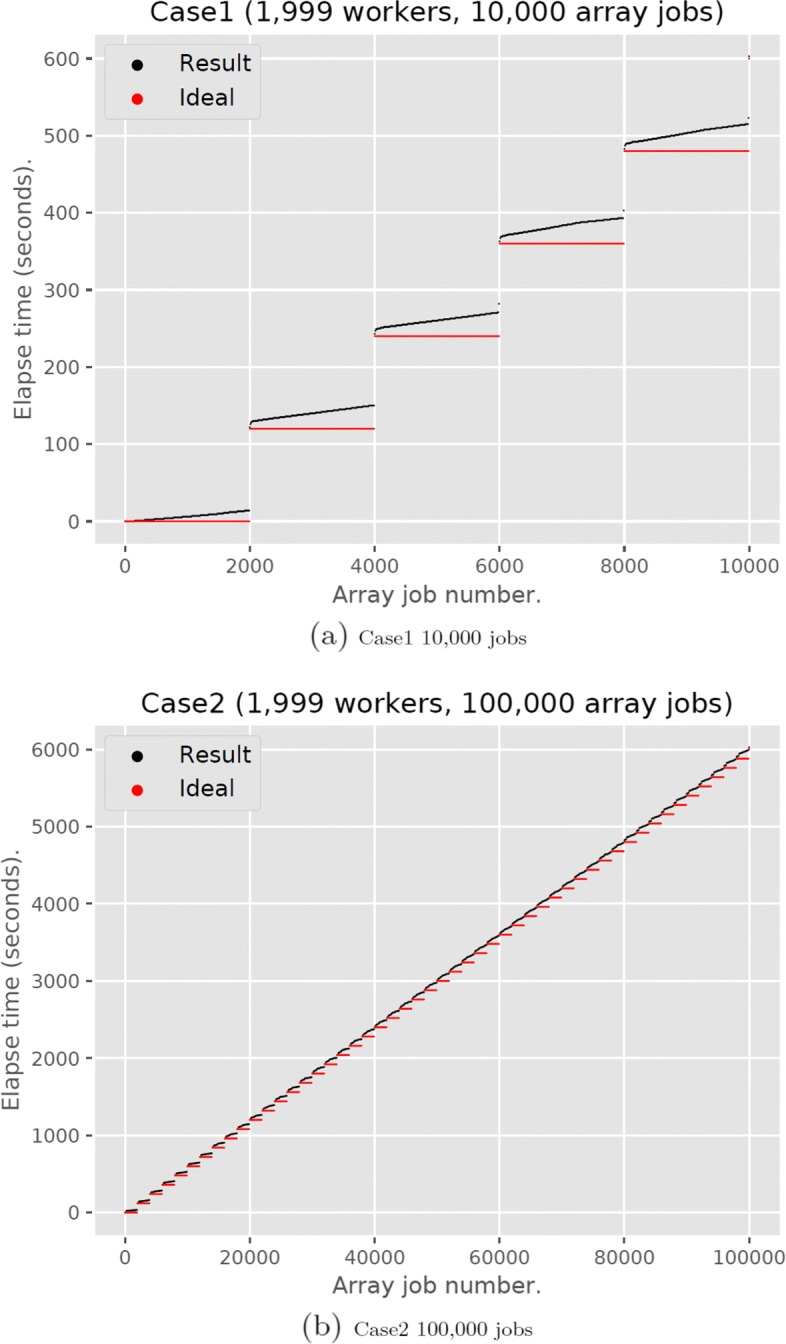
The results of VGE job assignments. (**a**) Case1 10,000 jobs (**b**) Case2 100,000 jobs

Figure 2 shows the start time of each job. In Case 1 (Fig. 2), workers executed five or six jobs. The red points indicate an ideal situation where the overhead is equal to zero. It is clear that the result in this figure shows this step, so the VGE master smoothly assigned jobs to workers.

The larger the number of jobs, the bigger the overhead. In Case 2 (Fig. 2), the number of jobs was ten times of Case 1, but the assignment was still performed smoothly. In Case 2, the ideal elapsed time for execution was 6120 seconds and the measured time for execution was 6145 seconds. Thus, the total VGE overhead was approximately 24.6 seconds and the assignment overhead for one job to a worker was 246 microseconds. This is sufficiently small to handle a large number of jobs.

### Simultaneous analyses of many samples

In this test, we focused on the massively parallel analysis of multiple samples simultaneously. The numerical environment was the K computer, the same as in the previous section. The test code included two tasks, the split and BWA [18] alignment of input FASTQ data [19], which is standard in high-throughput sequencer data analysis. The details of the input data are shown in Table 2. Here, we used 25,055 nodes (200,440 cores) for this calculation and accomplished it in three hours.

**Table 2:** Details of sample data

Number of samples	14
Data type	Whole-genome sequencing (WGS)
Data format	FASTQ
Read length	152 bp, paired-end
Total size	4.2 TByte, 3.8 Tbp

This result indicates that VGE has enough capability for controlling thousands of workers and handling multiple samples simultaneously.

## Discussion

### Performance and usability

We defined four requirements of VGE in “Goal and requirements” section. The first requirement (Non-privilege server program) has been described in “Algorithms” and “Results” section. Thus we focused on the other requirements in this part.

**Fig. 3:**
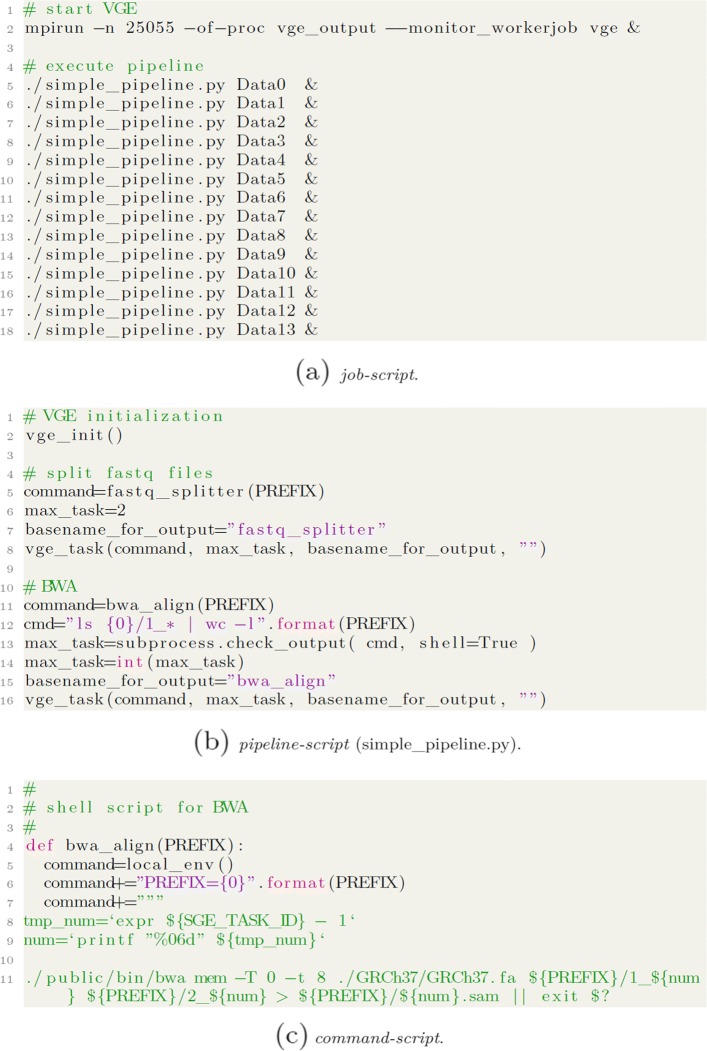
Sources of the scripts used for multiple sample analyses (short extraction). (**a**) job-script. (**b**) pipeline-script (simple_pipeline.py). (**c**) command-script

The second requirement is handling multiple tasks. Figure 3a shows a short extraction of the job-submitting script used in “Simultaneous analyses of many samples” section. Fourteen samples were named Data 0 to Data 13, respectively, and their tasks of FASTQ data division and BWA alignment were written in simple_pipeline.py.

A line starting with “simple_pipeline.py” corresponded to one sample analysis. In this example, fourteen sample jobs were submitted to VGE independently. In this way, VGE accepts multiple job submissions at once. Of course, different pipelines can also be submitted simultaneously.

Here, we focus on the results of job assignment and filling to workers. We used the same pipeline and data used in “Simultaneous analyses of many samples” section. The only difference was the number of workers. In this case, we used 5,000 workers which was much less than the number of total jobs. Thus, the workers had to perform the assigned jobs many times.

**Fig. 4:**
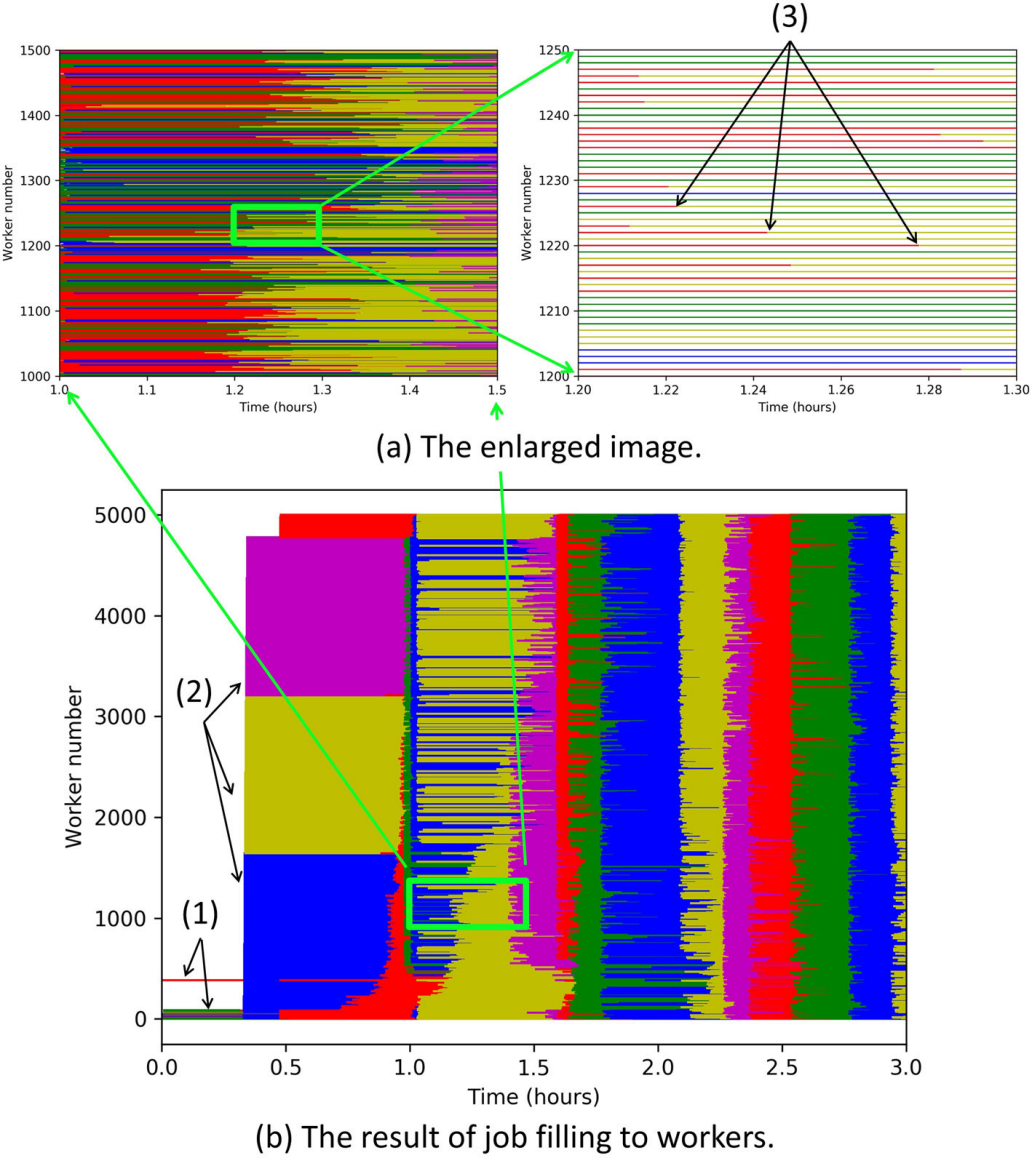
The results of filling jobs to workers. The colored bars mean that workers performed assigned jobs. The white space means workers waited for assigned jobs. Workers that performed jobs from a coinciding task were given the same color. Colors were used cyclically. The number of workers was 5,000. Figure (**a**) is an enlarged image of Figure (**b**) framed by a light green box. (1) Workers performed *fastq_splitter* tasks. The number of these tasks were 28, so that most of workers were sleepingt. (2) The first three tasks of *bwa_align* were assigned to workers and started at almost the same time. There were still sleeping workers because the other *fastq_splitter* had been calculating. (3) These workers first performed jobs belonging to a red-colored task and accomplished them. Then, they immediately started the next jobs belonging to a yellow-colored task. The complicated results shown in Figure (a) indicate that each worker worked independently and continuously despite the computational costs of each task being quite different

Figure 4 shows how the workers executed the jobs based on time. In the first twenty minutes, only a few workers performed jobs and the majority of the others did not work. This is because fourteen pipelines submitted *fastq_splitter*, which contained only two jobs. The results indicate that VGE successfully handled the dependency between tasks.

After this task, the FASTQ files were split into thousands of files that were aligned with BWA by all the workers. The number of split files was much larger than that of workers, so all workers continued to perform their jobs. From this figure, it can be concluded that the assignment was tightly arranged; thus, job management of VGE was very effective in a real case.

The third requirement is dependency control among tasks. Python is an interpreter language and performs a process per line. Using its characteristics, VGE controls task dependencies by tuning the task writing order in a script.

Figure 3 shows a short extraction of simple_pipeline.py used in “Simultaneous analyses of many samples” section. It consists of two tasks, the division of input FASTQ files (*fastq_splitter*) and alignment of decomposed files by BWA (*bwa_align*). Here, *bwa_align* must wait for completion of *fastq_splitter* task.

As described in “Algorithms” section, job submission to VGE is performed by using *vge_task()* function. It is clear from Fig. 3 that the simple_pipeline.py contains two tasks; the former is *fastq_splitter* and the latter is *bwa_align*.

The *vge_task()* written on the eighth line in Fig. 3 handles *fastq_splitter* task, and the process is accomplished after finishing the division of FASTQ files by VGE workers. Therefore, the *vge_task()* that is written later and corresponds to *bwa_align* does not submit to VGE before its accomplishment of the first task. The dependencies among tasks will be controlled by the order of tasks written in a script in this manner.

The final requirement is friendliness to medical and bioinformatics researchers. Pipelines using VGE consist of three parts: describing the concrete contents of tasks (hereinafter referred to as *command-script*), denoting the flow of the pipelines (*pipeline-script*), and submitting tasks to VGE (*job-script*) (Fig. 3).

The *command-script* and the *pipeline-script* can be written either in the same file or in independent files. The *command-script* can also be described in shell script. Therefore, legacy scripts can be used on VGE. However, development of scripts from scratch is also possible as researchers in this field are familiar with coding in Python.

On the other hand, *pipeline-script* must be written in Python. However, only *vge_task()* needs to be described. *vge_task()* requires three arguments: COMMAND, MAX_TASK, and BASENAME_FOR_OUTPUT. These arguments indicate the task name defined in *command-script*, the number of workers neccesary for the task (in short, the number of array jobs), and the unique ID (arbitral strings) used for log files, respectively. The value assignments are very clear, as shown in Fig. 3 (5-7 lines).

As discussed, VGE is very straightforward and friendly software for users.

### Issues associated with distributed file systems

**Fig. 5:**
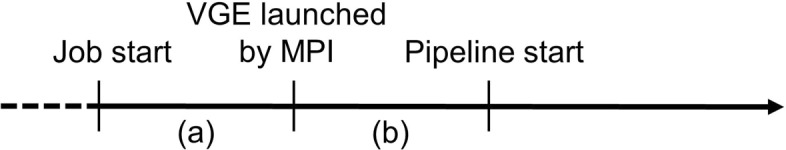
General behavior of a job using VGE

At the test described in “Simultaneous analyses of many samples” section, we encountered an unexpected severe problem. General behavior of a job using VGE is shown in Fig. 5. There are two intervals before the pipeline starts. First one is the initialization time of the system such as environmental value settings (a), and the other is the initialization time of MPI and VGE. Both intervals are from seconds to a minute in usual, but the total time of them was over 2 hours in the first trial.

This problem had observed first in this large-scale computation test, or it never appeared at smaller scale tests such as two thousand nodes. Therefore, VGE didn’t mainly cause this problem. We carefully investigated the cause of this problem with K computer operating team. According to the result of this investigation, both initialization intervals ((a) and (b)) took 1 hour respectively. In the system initialization interval (a), the operating system and JMS do various processes such as assigning of nodes, but we found that the file system became overload.

In this study, we mainly used the K computer that is one of the biggest supercomputers in the world. Of course, it equips a very large storage. Its size is over 30 PB thus traditional storage systems cannot handle such a huge storage. The K computer employs Fujitsu Exabyte File System [20] that was based on Lustre [21].

Lustre is one of distributed file systems. Lustre family file systems consist of three parts, one is physical disks, another is object storage servers (OSS), and the other is metadata servers (MDS). Thousands of physical disks and OSSs are used in Lustre family system, but the number of MDS is usually small. Therefore, MDS may become a bottleneck of Lustre family systems.

According to the investigation, we found that the job sent too much requests (e.g., make files, remove files, etc.) to the MDSs of FEFS at VGE launching. The observed value was over 20,000 per second. Applicable value of request to MDSs is 1300 per second, so that it was an extremely high value. The requests was caused by making log files of each workers. VGE workers make each log files in which the received task information and worker status are stored. The number of these files is proportional to the number of workers, thus we can’t find this problem in the previous tests. To avoid this problem, we made log files for VGE workers using only 1 process before MPI launched VGE.

**Fig. 6:**
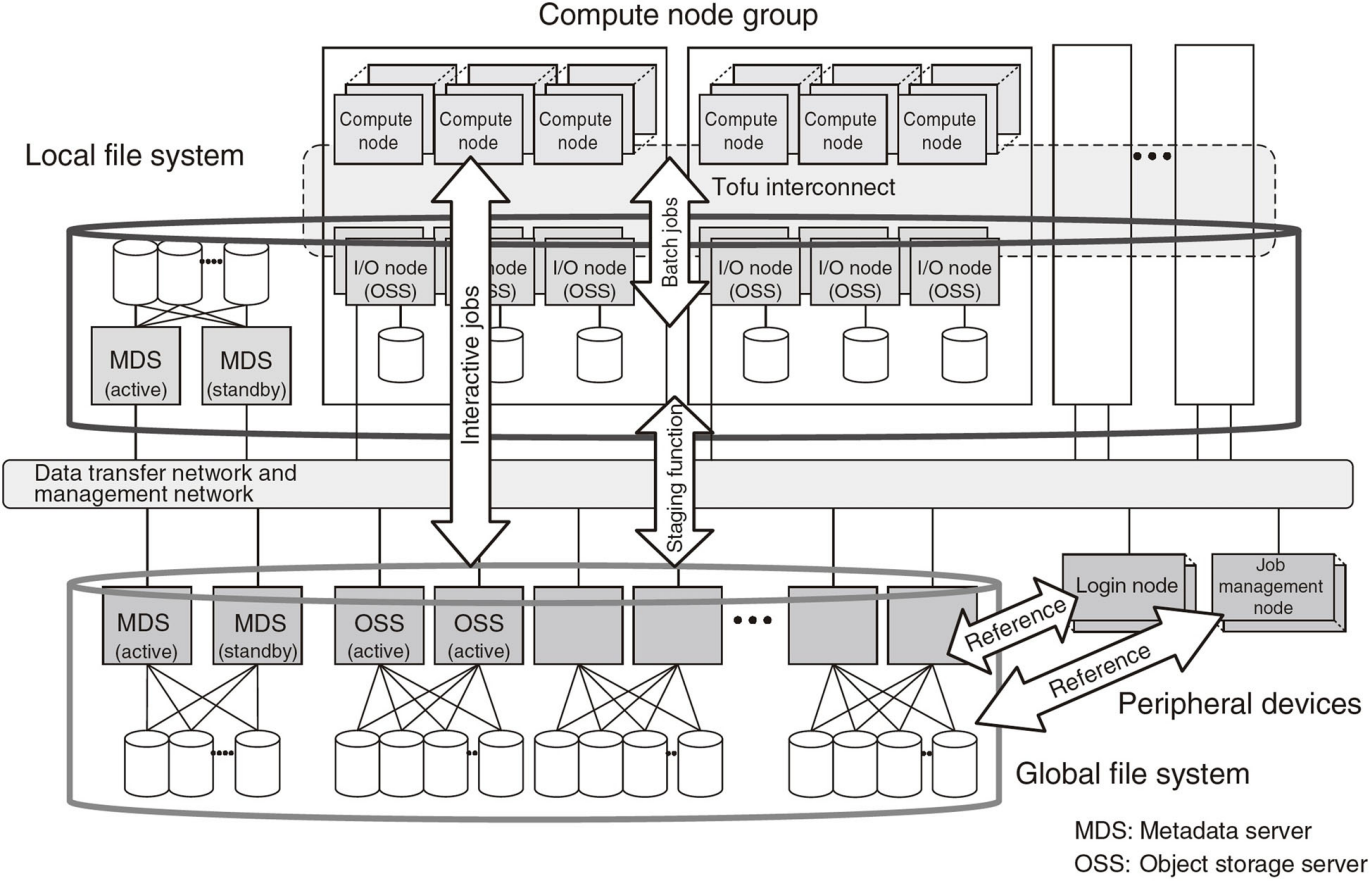
Fujitsu Exabyte File System (FEFS) [20]

Figure 6 shows FEFS structure in the K computer. The unique characteristics is that the whole file system consists of two layer: Global file system (GFS) and Local file system (LFS). Each system is complete as an independent file system. Programs and data that is required for a job send from GFS to LFS by the job management system (staging function) through the data transfer network. Thus user jobs are not affected by the others miscellaneous works on the login nodes.

The initialization time of MPI and VGE (b) was also related to MDS. In this initialization period, the system proceeds MPI startup and loads python modules that VGE imports. Each MPI process loads python module files respectively so that the requests to MDSs become very high in large scale tests. This problem is widely known in the dynamic linking library research field [22]. To avoid this problem, you may use tools that improve a library loading performance. In this study, we prepared python main system and all modules on the local disks of all calculation nodes. Since VGE master and workers didn’t access to the files of python on the FEFS, we reduced the number of access to the MDSs at this initialization intervals.

These issues were occurred in the large-scale computation of multiple sample analysis, so that you may consider that it is a very particular situation. However, it may occur in all types of bioinformatics analysis. As described before, one sample data size become quite large and it has already become over 5TB in the state-of-the-art studies. In such cases, typical protocols of sequence analysis hold potential risks for file systems.

## Conclusion

In this study, we developed MPI-based middleware named *Virtual Grid Engine* (VGE), that employs the Master-Worker algorithm and provides grid engine services. It achieved extremely low overhead costs in large-scale computation. In the test calculation on the K computer, we accomplished alignments of 4.2 TB, 3.7 Tbp FASTQ data in three hours, and the results indicate that this will contribute to the rapid analysis of multiple large-scale samples. We found problems related to distributed file systems in the large scale computation. These problems are usually hard to recognize and solve for bioinformaticians. We successfully overcame them by collaborating with the K computer operating team.

## Availability and requirements

**Project name:** Virtual Grid Engine **Project home page:**https://github.com/SatoshiITO/VGE**Operating system(s):** Linux **Programming language:** Python **Other requirements:** MPI4PY 2.0.0 or higher, MPICH or OpenMPI 2.0 or higher**License:** MIT license**Any restrictions to use by non-academics:** no

## Data Availability

VGE is freely available from https://github.com/SatoshiITO/VGE

## Abbreviations

FEFS: Fujitsu exabyte file system
GE: Grid engine
GFS: Global file system
JMS: Job management system
LFS: Local file system
MDS: Meta data server
NGS: Next-generation sequencer
OSS: Object storage server
VGE: Virtual grid engine
